# Characterization of a midgut mucin-like glycoconjugate of *Lutzomyia longipalpis* with a potential role in *Leishmania* attachment

**DOI:** 10.1186/s13071-016-1695-y

**Published:** 2016-07-25

**Authors:** Jitka Myšková, Anna Dostálová, Lucie Pěničková, Petr Halada, Paul A. Bates, Petr Volf

**Affiliations:** 1 Department of Parasitology, Faculty of Science, Charles University, Viničná 7, 128 44 Prague 2, Czech Republic; 2 Institute of Microbiology of the ASCR, v.v.i., Videňská 1083, 142 20 Prague 4, Czech Republic; 3 Division of Biomedical and Life Sciences, Faculty of Health and Medicine, Lancaster University, Lancaster, UK

**Keywords:** Phlebotomine sand flies, *Leishmania*, Lipophosphoglycan, Glycoprotein

## Abstract

**Background:**

*Leishmania* parasites are transmitted by phlebotomine sand flies and a crucial step in their life-cycle is the binding to the sand fly midgut. Laboratory studies on sand fly competence to *Leishmania* parasites suggest that the sand flies fall into two groups: several species are termed “specific/restricted” vectors that support the development of one *Leishmania* species only, while the others belong to so-called “permissive” vectors susceptible to a wide range of *Leishmania* species. In a previous study we revealed a correlation between specificity *vs* permissivity of the vector and glycosylation of its midgut proteins. *Lutzomyia longipalpis* and other four permissive species tested possessed O-linked glycoproteins whereas none were detected in three specific vectors examined.

**Results:**

We used a combination of biochemical, molecular and parasitological approaches to characterize biochemical and biological properties of O-linked glycoprotein of *Lu. longipalpis*. Lectin blotting and mass spectrometry revealed that this molecule with an apparent molecular weight about 45–50 kDa corresponds to a putative 19 kDa protein with unknown function detected in a midgut cDNA library of *Lu. longipalpis.* We produced a recombinant glycoprotein rLuloG with molecular weight around 45 kDa. Anti-rLuloG antibodies localize the native glycoprotein on epithelial midgut surface of *Lu. longipalpis*. Although we could not prove involvement of LuloG in *Leishmania* attachment by blocking the native protein with anti-rLuloG during sand fly infections, we demonstrated strong binding of rLuloG to whole surface of *Leishmania* promastigotes.

**Conclusions:**

We characterized a novel O-glycoprotein from sand fly *Lutzomyia longipalpis*. It has mucin-like properties and is localized on the luminal side of the midgut epithelium. Recombinant form of the protein binds to *Leishmania* parasites in vitro. We propose a role of this molecule in *Leishmania* attachment to sand fly midgut.

## Background

*Leishmania* protozoans, the causative agents of leishmaniases, are transmitted by bites of female phlebotomine sand flies. In the sand fly vector, *Leishmania* parasites must overcome various barriers to generate transmissible infections and ensure continuation of the life-cycle (reviewed by [1]). A crucial step in the vector phase of the *Leishmania* life-cycle is the binding of promastigotes to the sand fly midgut. Promastigotes insert their flagella into the microvillar border of the midgut epithelium and anchor themselves to the midgut surface [2, 3]. This attachment enables them to persist in the sand fly gut when the blood meal remnants are defecated.

A series of studies on the parasite-vector combination *Leishmania major*-*Phlebotomus papatasi* established an important paradigm; galactose residues on mono-galactosylated phosphodisaccharide repeats of the major surface glycoconjugate lipophosphoglycan (LPG) have been shown to bind to a galectin located on the surface of the sand fly midgut [4–7]. However, a more recent study reported partial involvement of the flagellar protein FLAG1/SMP1 in attachment of *L. major* to the midgut epithelium of *P. papatasi* [8], and earlier experiments with LPG-deficient mutants revealed that LPG is not required for the *Leishmania* attachment in many other sand fly species [9–11]. These studies indicate that alternative attachment molecules can be involved in midgut binding in addition to LPG-galectin.

Laboratory studies on vector competence to *Leishmania* parasites suggest that sand flies fall into two broad groups. Three species, i.e. *P. papatasi*, *P. duboscqi* and *P. sergenti,* are termed “specific” or “restricted” vectors that support the development of one *Leishmania* species only (*L. major* and *L. tropica*, respectively). Other sand fly species, importantly including vectors of the visceralising parasites *L. donovani* and *L. infantum*, belong to so-called “permissive” vectors, which are susceptible for a wide range of *Leishmania* spp. In laboratory conditions *Leishmania* parasites are capable of developing in any permissive vector, if given the opportunity (reviewed by [1, 12]).

In a study exploring the molecular basis of vector competence Myšková and colleagues [9] revealed a remarkable correlation between specificity *vs* permissivity of the vector and the glycosylation of its midgut proteins. The *Helix pomatia* agglutinin (HPA), a lectin specific for terminal N-acetyl-galactosamine (GalNAc) present on O-linked glycoconjugates, bound to midgut proteins from permissive but not from specific vectors. All five permissive species tested possessed HPA positive bands, whereas none were detected in the three specific vectors examined [9, 10, 13]. These findings suggested a hypothesis for the role of O-linked glycoconjugates in *Leishmania* binding, which was supported by two further observations. Fluorescent-labelled HPA (FITC-HPA) showed specific localisation of O-glycosylated epitopes in the microvillar border of sand fly midgut, the location required for *Leishmania* attachment, and the O-linked glycoconjugates recognised by FITC-HPA bound to the surface of *Leishmania* promastigotes [9].

In the present work we describe an O-linked glycoconjugate of the permissive vector *Lu. longipalpis* and show that it has mucin-like properties. Functional testing was performed using the recombinant form of the *Lu. longipalpis* glycoconjugate (rLuloG) and specific anti-rLuloG antibodies using in vivo and in vitro methods. For in vivo studies, experimental infections of *Lu. longipalpis* with *L. infantum* were performed as described previously for *L. major* and *P. papatasi* galectin [6]: *L. infantum* parasites were mixed with blood reconstituted with specific anti- rLuloG serum to test for blocking of parasite development in the sand fly midgut. In vitro experiments were performed to investigate the binding of rLuloG to *Leishmania* parasites.

## Methods

### Sand fly colonies and parasites

Laboratory colonies of two sand fly species were used: *Lutzomyia longipalpis* (origin from Jacobina, Brazil) and *Phlebotomus papatasi* (origin from Turkey). Colonies were maintained in conditions described previously [14] and fed on 50 % sucrose. Most experiments were done with 4–7 day-old females, and the expression of LuloG was compared in females aged from 1 to 12 days.

The following parasite isolates were used: *Leishmania infantum* (MHOM/BR/76/M4192) and *Leishmania major* LV561 (MHOM/IL/67/LRC-L137). Parasites were maintained at 26 °C on Medium 199 supplemented with 20 % foetal bovine serum (Thermo Fisher Scientific, Waltham, USA). Prior to sand fly infections, *Leishmania* parasites were washed by centrifugation and resuspended in saline.

### Detection of O-linked glycoconjugates in *Lutzomyia longipalpis* midguts

Female midguts were homogenized mechanically by repeated freezing and thawing, proteins were separated by SDS-PAGE (10 % gel, reducing conditions, 10 guts per lane), transferred to nitrocellulose and analyzed by lectin blotting. The nitrocellulose membrane was incubated with Tris-NaCl-Tween (20 mM Tris, 150 mM NaCl, pH 7.6) and blocked with 5 % (w/v) bovine serum albumin (BSA; Sigma-Aldrich, St. Louis, USA; diluted in Tris-NaCl-Tween) overnight at 4 °C and then for 1 h at room temperature with biotinylated *Helix pomatia* agglutinin (HPA; 1 μg/ml; Sigma-Aldrich). After repeated washing the blots were incubated with streptavidin peroxidase (2.5 μg/ml; Sigma-Aldrich, St. Louis, USA; in Tris-NaCl-Tween) and the peroxidase reaction was developed in 4-chloro-1-naphthol solution (Sigma-Aldrich). Experiments were done with females of various ages and physiological states (e.g. females which did not feed on blood, females after blood-feeding) as indicated.

### Characterization of the midgut glycoconjugate

Lysates were prepared from 20 midguts of *Lu. longipalpis* females. Triton X-114 was used for separation of hydrophilic and amphiphilic proteins as previously decribed by Bordier [15] with following modifications: the lysates were incubated with the 1 % Triton X-114 for 90 min on ice and the separation was done without sucrose cushion. Triton X-114 is a non-ionic detergent with a low cloud point (about 20–23 °C) enabling protein solubilization with phase-partitioning of hydrophilic from amphiphilic proteins. The aqueous and the detergent phase were then analyzed by SDS-PAGE and blotting with HPA (as decribed above). To study the presence of a glycophosphatidylinositol (GPI) anchor, lysates in Tris-NaCl were incubated for 1.5 h at 37 °C with protease inhibitors (Complete, Roche, Mannheim, Germany) and 1 unit phosphatidylinositol-specific phospholipase C (PI-PLC), a highly purified enzyme prepared from *Bacillus cereus* (Sigma-Aldrich). The sample was then fractionated with Triton X-114 and analyzed by SDS-PAGE followed by blotting with HPA.

To check the isoelectric point of the candidate glycoconjugate, a lysate of 50 midguts was analyzed by 2D electrophoresis (IEF/SDS-PAGE), followed by blotting with HPA incubation as described above.

To generate sufficient material for protein identification, HPA affinity chromatography was used to purify the O-linked glycoconjugate from midguts of *Lu. longipalpis* females. Lysates of 1400 midguts were incubated with 9 units of PI-PLC in Tris-NaCl and protease inhibitors (Complete, Roche) for 1.5 h at 37 °C and loaded onto a HPA-agarose column (Sigma-Aldrich). After washing, specifically bound molecules were eluted with 0.5 M GalNAc in Tris-NaCl and analyzed by SDS-PAGE (10 % gel, reducing conditions) or 2D electrophoresis (IEF/SDS-PAGE). The glycoconjugate band/spot was stained by Pro-Q-Emerald glycoprotein stain (Thermo Fisher Scientific), a technology available for detection of glycoconjugates in gels. The protein band/spot was excised from the gel, cut into small pieces and washed using 50 mM 4-ethylmorpholine acetate (pH 8.1) in 50 % acetonitrile (MeCN). The protein was reduced with 30 mM Tris(2-carboxyethyl)phosphine (TCEP) in 100 mM Tris-HCl (pH 8.0) at 65 °C for 30 min and alkylated by 30 mM iodacetamide in 100 mM Tris-HCl (pH 8.0) for 60 min in the dark. The gel pieces were then incubated overnight at 37 °C in a cleavage buffer containing 25 mM 4-ethylmorpholine acetate, 5 % MeCN and trypsin (100 ng; Promega, Woods, USA) or Asp-N protease (30 ng; Roche). The resulting peptides were extracted into 40 % MeCN/0.1 % TFA. One μl of the peptide mixture was deposited on a MALDI plate, allowed to air-dry at room temperature and overlaid with 0.4 μl of MALDI matrix (aqueous 50 % MeCN/0.1 % TFA solution of α-cyano-4-hydroxycinnamic acid; 5 mg/ml; Sigma-Aldrich). Mass spectra were measured on an Ultraflex III MALDI-TOF instrument (Bruker Daltonics, Bremen, Germany) equipped with LIFT technology for MS/MS analysis. MS data were searched using an in-house MASCOT search engine against the NCBI nr database subset of eukaryotic proteins.

### Expression and purification of rLuloG in H5 insect cells

RNA was isolated from 10 midguts of *Lutzomyia longipalpis* females using a High Pure RNA isolation kit (Roche) and reverse-transcribed using Superscript III reverse transcriptase (Thermo Fisher Scientific) following the manufacturer’s instructions. The sequence of the cDNA corresponding to transcript GenBank: EU124597 was amplified using Q5 High Fidelity DNA polymerase (BioLabs, Ipswich, USA) (forward primer: 5′-CTT AAA TGC TAC AAT TGC AAT TCC T-3′ and reverse primer: 5′-ACT ACT CTC AGT TGT AGT TGG A-3′) omitting the predicted signal peptide (SignalP, Lyngby, Denmark) and GPI anchoring signal (PredGPI, gpcr2.biocomp.unibo.it). The resulting amplicon was cloned into pFastBac HBM TOPO vector and expressed in High Five *Trichoplusia ni* cells using the Bac-to-Bac HBM TOPO secreted expression system (Thermo Fisher Scientific) following the manufacturer’s instructions. Infected High Five cells were scraped from Petri dishes and transfered to a microcentrifuge tube. The solution was centrifuged first at 1500 rpm for 7 min and the supernatant was concentrated using 30 kDa filters Amicon Ultra-15 (Millipore, Carrigtwohill, Ireland), according to the manufacturer’s instructions. The resulting sample was incubated with HPA agarose (Sigma-Aldrich) for 2 h. The recombinant protein named rLuloG was specifically eluted with 0.5 M GalNAc in Tris-NaCl and washed several times in Tris-NaCl on 30 kDa filters. The purity and molecular weight was confirmed by SDS-PAGE (10 % gel, reducing conditions) followed by Coomassie blue staining and blotting with HPA (as described above). The band was excised from the gel, digested by trypsin and analyzed by MALDI-TOF mass spectrometry as described above.

### Rabbit anti-rLuloG antibodies: western blotting and indirect immunofluorescence with *Lu. longipalpis* midguts

Polyclonal anti-rLuloG antibodies were commercially prepared by repeated immunization of rabbit with 2 mg of purified rLuloG (Eurogentec, Seraing, Belgium). Antibody binding to rLuloG and to native protein from *Lu. longipalpis* midguts (10 guts per lane) was analyzed by SDS-PAGE (10 % gel, reducing conditions) followed by western blotting. The nitrocellulose membrane was incubated with Tris-NaCl-Tween (20 mM Tris, 150 mM NaCl, pH 7.6) and blocked with 6 % milk diluted in PBS/Tween overnight at 4 °C. The membrane washed with PBS/Tween was incubated first for 1 h with anti-rLuloG serum (1: 15,000 in 3 % BSA in PBS/Tween), repeatedly washed and then incubated with peroxidase-conjugated anti rabbit IgG SwAR/Px (Sevapharma a.s., Prague, Czech Republic), 1:1000 in 3 % BSA in PBS/Tween, as the secondary antibodies. After repeated washing the colour reaction was developed in 4-chloro-1-naphthol solution. Preimmune rabbit sera or midguts of the specific vector *P. papatasi* were used as negative controls. Incubation of separated rLuloG with HPA and inhibiton of this reaction with specific GalNAc were also tested.

Anti-rLuloG antibodies were also used to localize LuloG in sand fly midguts. To mimic the natural situation during *Leishmania* infections, midguts were dissected from *Lu. longipalpis* females which had defecated blood meal remnants (4–6 days post-blood meal). Midguts were longitudinally opened, fixed in 2 % paraformaldehyde for 20 min, washed twice in Grace’s Insect Medium (GIM, Sigma-Aldrich) and incubated with preimunne serum or anti-rLuloG antibodies (1:300 in 1 % BSA in GIM) for 1 h in RT. Guts were then washed four times in GIM and incubated with FITC anti-rabbit IgG (1: 50 in 1 % BSA in GIM) for 1 h. After washing in GIM, guts were placed in 2 μl of cooled CyGel (Biostatus Limited, Leicestershire, UK) on a microscope slide, carefully flattened, mounted in Prolong Gold antifade reagent (Thermo Fisher Scientific) and examined under a fluorescent microscope Olympus BX51.

### Binding of rLuloG to *Leishmania* promastigotes

A 3-day culture of *L. major* LV561 was washed by centrifugation with PBS, fixed with 2 % formaldehyde for 30 min, washed again, diluted to a final density of 10^7^ promastigotes/ml, spotted onto a slide and air-dried. Fixed parasite cells were rehydrated in PBS for 5 min, blocked with 0.1 % BSA in PBS for 5 min and incubated with rLuloG for 1 h. Slides incubated with preimunne serum or 0.1 % BSA served as negative controls. After washing (PBS for 15 min) the cells were incubated first with anti-rLuloG serum (1:500 in 0.1 % BSA in PBS) for 1 h, washed and then incubated with ALEXA fluor 488 goat anti-rabbit IgG (Thermo Fisher Scientific; 1:500 in 0.1 % BSA in PBS) in the dark. Slides were dried and mounted in Prolong Gold antifade mounting medium (Thermo Fisher Scientific) and examined under the fluorescent microscope Olympus BX51.

### *Leishmania* development in *Lu. longipalpis*: effect of anti-rLuloG serum

Sand fly females were infected by feeding through a chick skin membrane with *L. infantum* promastigotes from 3-day-old cultures (cell density of 5 × 10^5^/ml) in heat-inactivated rabbit blood (Bioveta, Ivanovice na Hané, Czech Republic) reconstituted either with anti-rLuloG serum or control preimmune serum. Blood-engorged females were separated and maintained at 26 °C. On day 2 post-blood meal (PBM) (i.e. before defecation) or day 4 and 7 PBM (i.e. after defecation of blood meal remnants) the females were dissected for microscopical examination.

Parasite density in dissected midguts was graded according to criteria reported previously as light (< 100 parasites/gut), moderate (100–1000 parasites/gut) or heavy (> 1000 parasites/gut) [16]. Experiments were repeated three times. Infection rates (number of infected *vs* uninfected females) and intensities of infection (heavy, moderate, light, zero) were compared between groups using Chi-square test and S-PLUS 2000 programme.

## Results

### Detection and characterization of midgut glycoconjugate

**Fig. 1:**
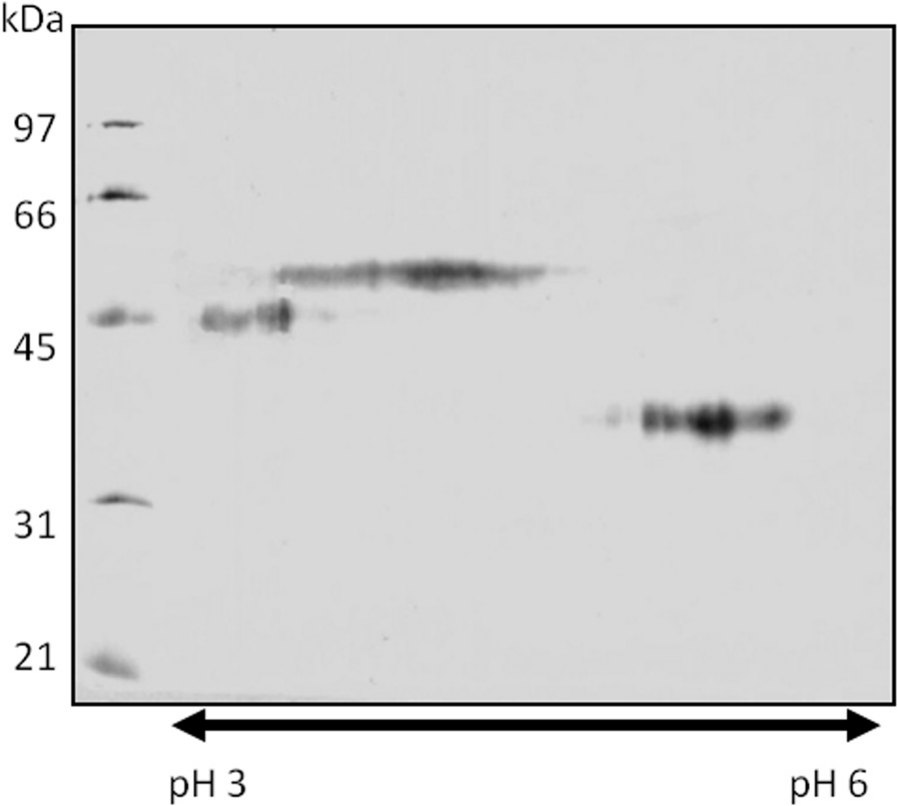
Two-dimensional gel electrophoresis showing the acidic feature of Lulo protein. Homogenate of 50 *Lutzomyia longipalpis* midguts was separated in 2-DE electrophoresis and GalNAc-containing glycoconjugates were detected by western blotting with lectin from *Helix pomatia* (HPA)

**Fig. 2:**
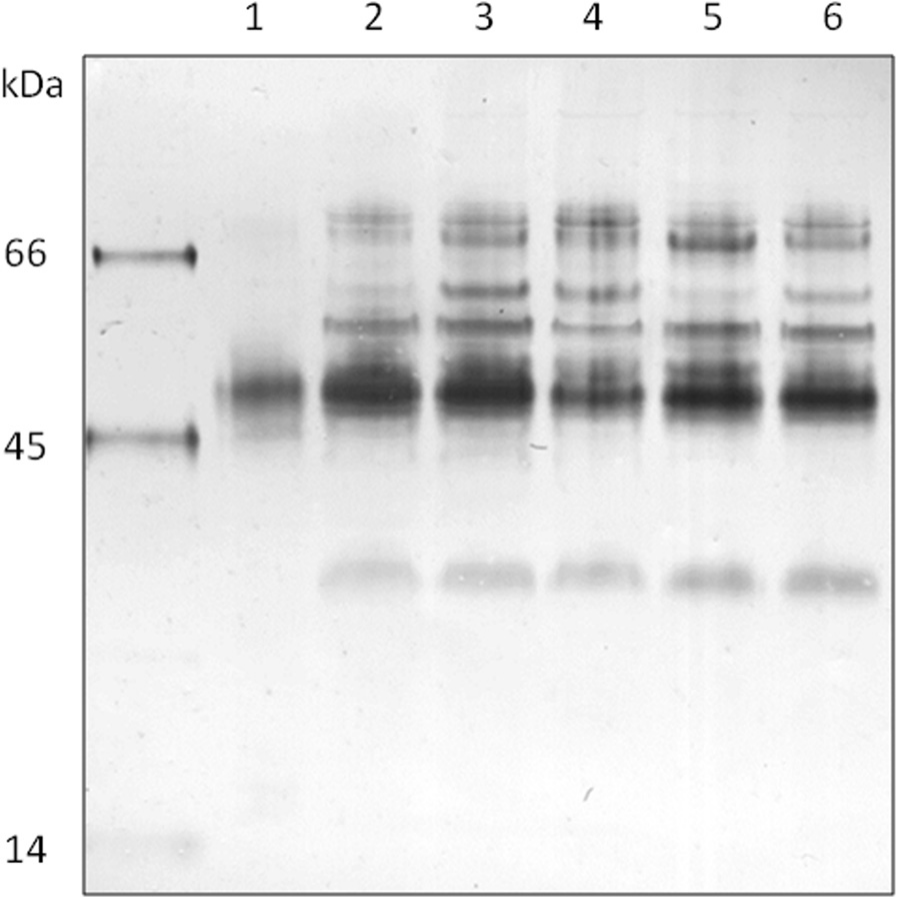
Detection of the GalNAc-containing glycoconjugate during the life of adult females. Homogenate of ten *Lu. longipalpis midgut* was separated by SDS-PAGE followed by western blot with lectin from *Helix pomatia* (HPA). Lane 1: 1-day-old females; Lane 2: 4-day-old females; Lane 3: 8-day-old females; Lane 4: 8-day-old females after blood meal; Lane 5: 12-day-old females; Lane 6: 12-day-old females after blood meal

Midgut lysates of the permissive vector *Lu. longipalpis* were analyzed by SDS-PAGE, followed by blotting with *Helix pomatia* agglutinin. This lectin is specific for GalNAc, a carbohydrate typically associated with O-linked glycan-displaying glycoconjugates such as mucins. HPA showed reactivity with a molecule of around 45–50 kDa molecular mass; under reducing SDS-PAGE we also detected one additional band around 35 kDa. These sand fly O-linked glycoconjugates do not resolve into sharp bands under SDS-PAGE as commonly found for heavily glycosylated proteins, and are relatively acidic with pI values from 3 to 5, as indicated by IEF/SDS-PAGE (Fig. 1). The glycoconjugate was detectable during the whole life-cycle of adult females, i.e. after hatching, before and after blood-feeding (Fig. 2).

**Fig. 3:**
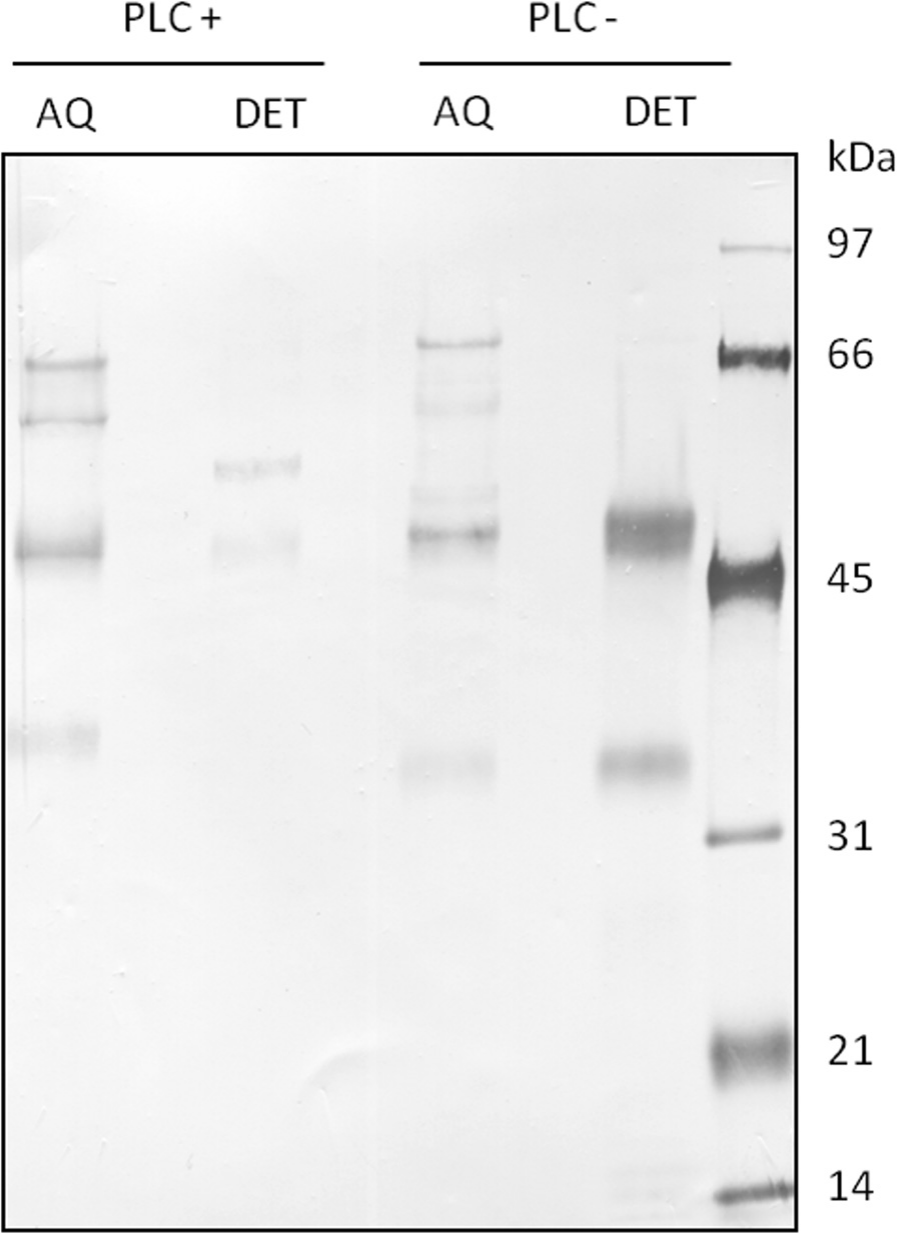
GalNAc-containing glycoconjugates are sensitive to phosphatidylinositol-specific phospholipase C (PI-PLC) exposure. Lysate of *Lu. longipalpis* midguts was fractionated by Triton X-114 and analyzed by SDS-PAGE and western blot with lectin from *Helix pomatia* (HPA). HPA-binding molecules were detected mostly in the detergent phase (DET PLC-) and partially in the aqueous phase (AQ PLC-). In contrast, the HPA-binding molecule in midgut lysate after exposition to PI-PLC was detected mostly in the aqueous phase (AQ PLC+) and not in the detergent phase (DET PLC+)

Extraction of sand fly midgut membranes with Triton X-114 before PI-PLC treatment recovered the HPA binding molecule in the detergent-rich phase, whereas after PI-PLC treatment most of the glyconconjugate was present in the aqueous phase (Fig. 3). These results are clearly indicative of a GPI membrane anchor for the glycoconjugate.

**Fig. 4:**
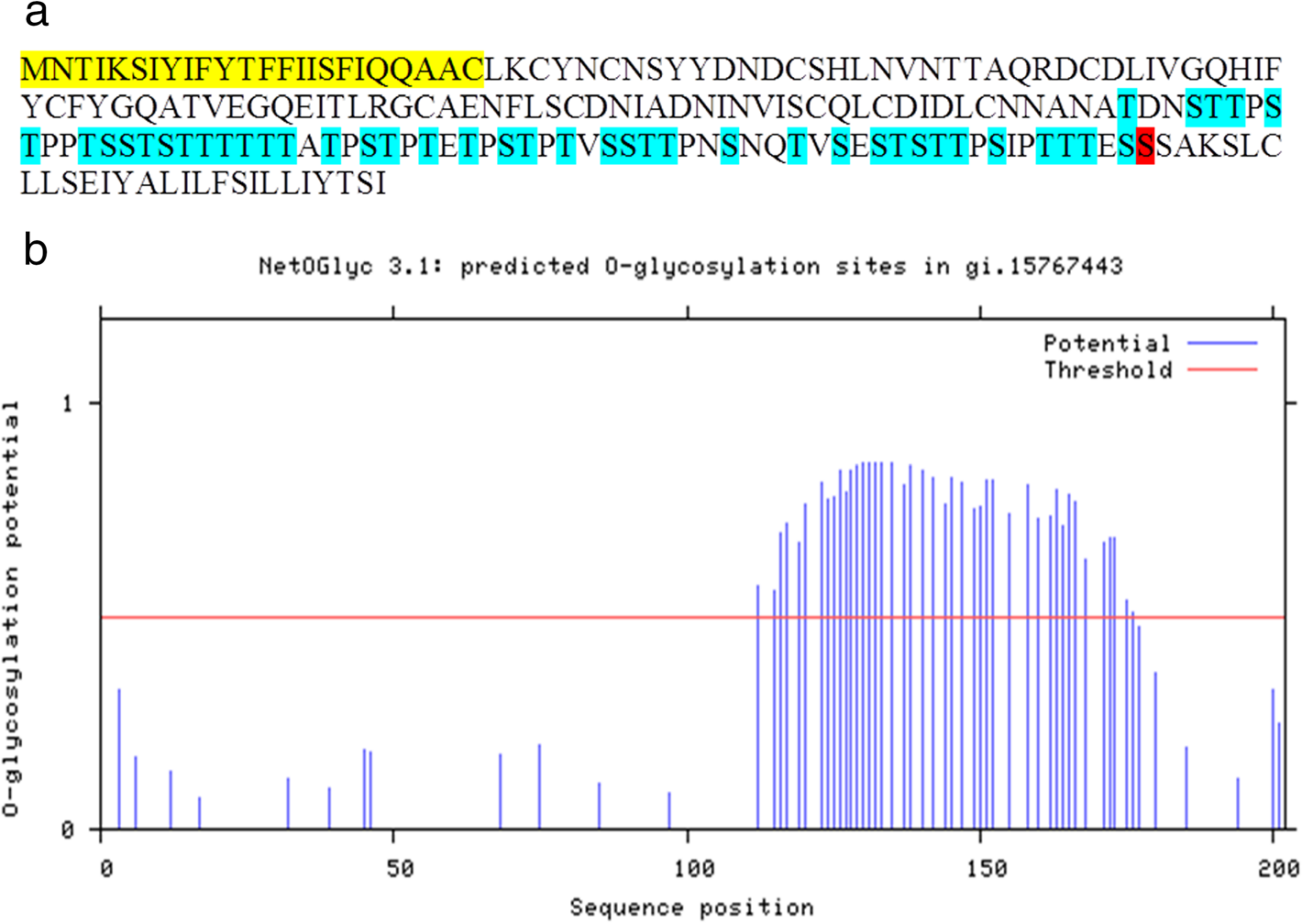
Lulo protein sequence with C-terminal part rich in potentially O-glycosylated Ser/Thr residues **a** Highlighted in *blue* are potentially O-glycosylated Ser/Thr residues (NetOGlyc: http://www.cbs.dtu.dk/services/NetOGlyc/); highlighted in *yellow* is a signal peptid (http://www.cbs.dtu.dk/services/SignalP/); site highlighted in *red* is the GPI-anchor predicted place (http://gpcr2.biocomp.unibo.it/gpipe/index.htm) **b** Predicted O-glycosylation sites; blue lines going above the treshold line (red) show potential O-glycosylated sites

The PI-PLC-treated sample was subjected to HPA-affinity chromatography and the eluted fraction separated by SDS-PAGE. Mass spectrometry of the glycoconjugate spot identified peptides resulting from both trypsin- and AspN-digestion (YGQATVEGQEITLR and DLIVGQHIFY, respectively) corresponding to a *Lutzomyia longipalpis* 19 kDa midgut protein (GenBank: ABV60315). The C-terminal part of the protein sequence is rich in potentially O-glycosylated Ser/Thr residues (NetOGlyc prediction), forming a mucin-like domain (Fig. 4). This is in agreement with the observed lectin-binding properties of this glycoconjugate. Moreover, the high number of carbohydrate residues likely account for the majority of the apparent molecular weight of the molecule.

### rLuloG, anti-rLuloG and their binding to *Leishmania* promastigotes and sand fly midguts

**Fig. 5:**
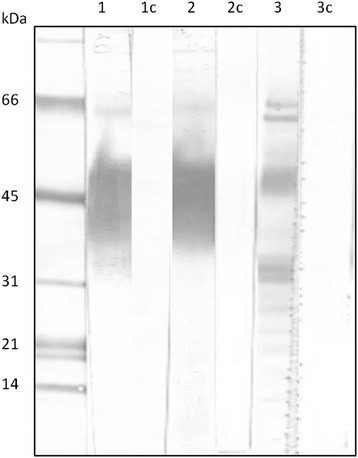
Recombinant Lulo protein has a molecular weight around 40–45 kDa and is recognized by anti-rLulo antibodies. The recombinant Lulo protein was purified by affinity chromatography with lectin from *Helix pomatia* (HPA). Eluent together with lysate of *Lu. longipalpis* midguts was analyzed by SDS-PAGE, followed by western blot with anti-rLuloG antibodies. Antibodies against rLuloG recognized one molecule in fraction eluted with N-acetyl-galactosamine (GalNAc), which has molecular weight around 40–45 kDa (Lane 1). Preimunne serum was used as a control, which did not recognize any molecules in fraction eluted with GalNAc (Lane 1c). The recombinant molecule was labeled also with HPA (Lane 2) and the reaction was inhibited by specific GalNAc (Lane 2c). Anti-rLulo serum recognized also the native molecule in a midgut lysate of *Lu. longipalpis* (Line 3), preimunne serum did not react with the *Lu. longipalpis* midgut lysate (Lane 3c)

Constrained by the small amount of the native molecule available, we opted for producing a recombinant version, in order to be able to produce antibodies specifically recognizing the identified glycoconjugate. We used the baculovirus secreted expression system in High Five insect cells, which allows for expression of glycosylated proteins. The purified recombinant rLuloG was subjected to SDS-PAGE, followed by western blotting and compared to the native mucin-like molecule (Fig. 5). rLuloG had a molecular weight around 40–45 kDa and did not resolve into sharp bands, similar to the native midgut protein. The identity of rLuloG was confirmed by MALDI-TOF mass spectrometry yielding 13 tryptic peptides covering 59 % of rLuloG sequence. Rabbit antiserum raised against purified rLuloG was found to strongly recognize the native glycoconjugate from *Lu. longipalpis* midguts as well the recombinant molecule. The lectin HPA recognized the same protein band, no reaction was found with preimunne serum (Fig. 5).

**Fig. 6:**
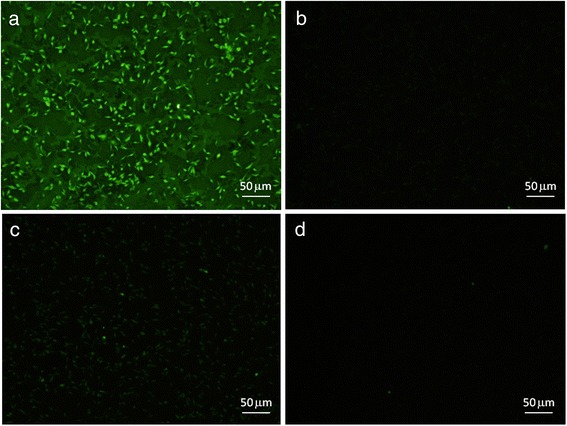
Binding of recombinant Lulo protein to *Leishmania major* promastigotes. Promastigotes were fixed on slides and incubated with recombinant Lulo protein, followed by anti-rLulo serum incubation and Alexa-anti rabbit IgG labelling. The entire body, including the flagellum was brightly stained by green fluorescent staining (**a**). In contrast, parasites incubated with preimunne serum instead of anti-rLulo serum were not stained with Alexa-anti rabbit IgG (**b**). In the negative controls, parasites incubated with BSA instead of rLulo protein, then with anti-rLulo serum and followed by Alexa staining were stained only weakly (**c**) and parasites incubated only with Alexa fluor labelling were not stained at all (**d**)

To further investigate the role of the *Lu. longipalpis* glyconconjugate in *Leishmania* attachment, parasites were incubated with recombinant Lulo protein, followed by anti-rLuloG serum incubation and Alexa-anti rabbit IgG labelling. Results showed that the rLuloG protein specifically bound to the surface of *Leishmania*, since the whole cell body, including the flagellum was brightly stained by green fluorescent staining (Fig. 6). In contrast, parasites incubated with preimunne serum instead of anti-rLuloG serum were not stained with Alexa-anti rabbit IgG. Lack of staining was observed also in other negative controls: parasites incubated with BSA instead of rLuloG protein, then with anti-rLuloG serum and followed by Alexa staining or parasites incubated with Alexa-anti rabbit IgG only (Fig. 6).

**Fig. 7:**
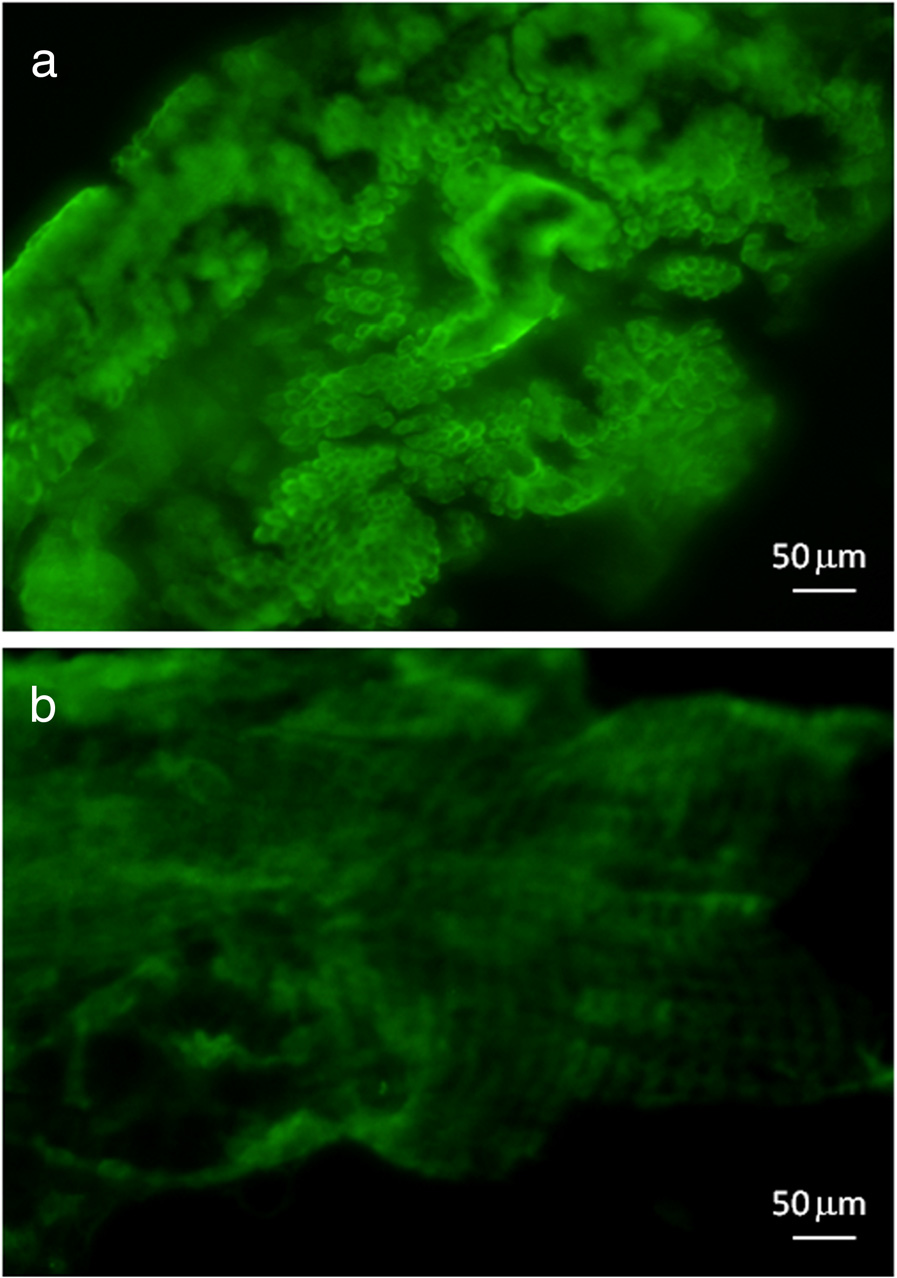
Binding of anti-rLulo antibodies to *Lutzomyia longipalpis* midgut epithelium. To investigate the distribution of GalNAc-displaying molecules across the midgut, midguts of *Lu. longipalpis* were fixed and incubated with either anti-rLulo sera (**a**) or preimunne sera (**b**) and labelled with FITC-anti rabbit IgG. Both groups of midguts were stained with similar intensity, but only in guts preincubated with anti-rLulo serum we observed staining of typical spherical/round epithelial cells, which are located on the luminal surface of midgut [17]

Anti-rLuloG antibodies were used to localize the *Lu. longipalpis* mucin-like glycoconjugate on dissected midguts by immunofluorescence. Midguts dissected from blood-fed and defecated *Lu. longipalpis* were longitudinally opened and incubated with preimmune serum or anti-rLuloG antibodies, followed by incubation with FITC-anti rabbit IgG. Midguts preincubated with preimmune serum were stained mainly nonspecifically on the outer surface where only muscle fibres are visible in the fluorescent microscope. Midguts preincubated with anti-rLuloG serum revealed strong reaction with the epithelial (inner) midgut surface composed of spherical/round epithelial cells, which are present only in the midgut luminal side [17] (Fig. 7).

### Development of *L. infantum* in *Lu. longipalpis* midgut fed with anti-rLuloG antibodies

**Fig. 8:**
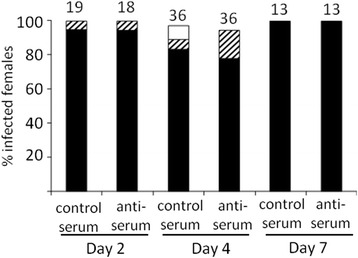
*In vivo* inhibition of *Leishmania chagasi* binding to *Lutzomyia longipalpis* midgut by anti-rLulo antibodies. *Lu. longipalpis* females were fed with *Leishmania chagasi* wild type in blood reconstituted with anti-rLulo serum (anti-serum) or normal rabbit blood (control serum). Day 2 dissections were before defecation, day 4 and 7 dissections were after defecation. Parasite loads were classified into three categories: heavy (more than 1000 parasites per gut; *black bars*); moderate (100–1000 parasites per gut; *striped bars*); light (1–100 parasites per gut; *white bars*). Numbers above the bars indicate the number of dissected females. On day 2 post-infection, both groups of sand flies initially produced heavy or moderate parasite loads in the abdominal midgut. However, after defecation on day 4, intensity of infection was slightly lower in sand flies fed on blood with anti-rLulo serum. In the following days, both groups developed uniformly, with heavy infection in all dissected guts. Data are from two representative experiments

In order to test whether the glycoconjugate from *Lu. longipalpis* midguts could be the ligand of the sand fly to which *Leishmania* parasites attach *in vivo*, blood-feeding experiments were performed, where the rabbit serum in blood was reconstituted with anti-rLuloG serum. On day 2 post-infection (PI), prior to defecation of the digested blood meal, there was no significant difference in the number and localization of parasites in the midgut of sand flies fed on blood with anti-rLuloG serum and in blood with normal rabbit serum. Very high infection rates (100 %) were present in both groups, with heavy infections in 98 % of dissected flies. On day 4 PI, when the blood meal was defecated, females infected with parasites mixed with normal serum retained a high infection rate (almost 100 %) and high parasite loads, with heavy infection in 83 % of flies. However, females infected with parasites mixed with anti-rLuloG serum showed a decrease in parasite loads, with heavy infection in 78 % of flies (Fig. 8). Although the inhibitory effect of anti-rLuloG antibodies on the parasite presence in the sand fly midgut was not statistically significant, there was a tendency to lower parasite numbers on day 4. It correlated with the time of *Leishmania* attachment to the sand fly midgut and subsequent defecation of blood meal remnants which occurs on days 3–5 days PI.

## Discussion

Data reported by Myšková and colleagues [9] suggested that a 45 kDa O-linked glycoprotein on the *Lu. longipalpis* midgut epithelium may function as a parasite ligand. Here we used a combination of biochemical, molecular and parasitological approaches to characterize the biological properties of this glycoconjugate. We provide evidence that the molecule with an apparent molecular weight 45 kDa on SDS-PAGE corresponds to a putative 19 kDa protein with unknown function detected in a midgut cDNA library of *Lu. longipalpis* [18]. The difference between the size of the native protein and predicted protein from cDNA library is assumed to be caused by a high degree of glycosylation. This is in agreement with the numerous predicted glycosylated serine and threonine residues found in the C- terminal part of the protein sequence, and also corresponds with the smeared appearance of the 45 kDa band on the gels or blots.

O-glycosylation prediction on the entire set of putative proteins identified in the midgut transcriptome of *Lutzomyia longipalpis* [18] did indeed find LuloG as the most heavily O-glycosylated protein. Beside LuloG, one more putative protein with extensive glycosylation was identified: ABV60335. It is a putatively secreted protein of 16.6 kDa with no other apparent conserved domains. No protein with a similar degree of O-glycosylation (a cluster of more than 40 putatively glycosylated Ser/Thr residues) was identified in the *P. papatasi* midgut transcriptome data [19].

An essential prerequisite for this GalNAc displaying molecule of permissive sand flies to function as a midgut receptor for *Leishmania* is its abundance and localization on midgut epithelial cells. Figure 7 shows that antibodies against the recombinant LuloG protein recognized round/spherical cells of dissected *Lu. longipalpis* midguts, typical of the luminal surface of epithelial midgut cells [17]. On the other hand, preimunne serum recognized mostly musculature, consisting of circular and longitudinal fibres, which is located on the external side of sand fly midgut [20].

Although we could not prove involvement of LuloG in *Leishmania* attachment by blocking of the native protein on the epithelial surface with anti-rLuloG during sand fly infections, we demonstrated a strong binding ability of rLuloG to whole *Leishmania* body by in vitro indirect imunofluorescence experiments. This suggests that the receptor for the sand fly glycoconjugate is localized on the entire surface of promastigotes, including the body and flagellum and is accessible to the sand fly midgut ligand. Our data correspond with the observations of Myšková and colleagues [9], where midgut lysates of the permissive sand fly *P. halepensis* bound to *Leishmania* promastigote surfaces, and this interaction was visualized by FITC labelled HPA, the lectin specific for GalNAc.

We can speculate about the reason for the in vivo blocking attachment results. First, anti-rLuloG antibodies could be proteolytically degraded during blood digestion in the sand fly midgut, which is commonly observed in oral immunotherapy used for mammals. Proteolytic enzymes involved in the degradation of orally administered immunoglobulins in humans include pepsin, trypsin, chymotrypsin, carboxypeptidase and elastase [21]. In *P. papatasi* and *Lu. longipalpis* midguts, trypsin-like proteases were described as the most abundant. Secondly, the effectivity of anti-rLuloG antibodies in *Leishmania* attachment could be limited due to the recombinant protein rLuloG being expressed in soluble form without a GPI anchor, which was used for antibody production. Important roles for GPI anchors in binding of antibodies was demonstrated by numerous studies where removal of the GPI lipid moiety can influence ligand binding properties, most likely due to conformational changes in the protein. Studies showed that antibodies raised against the soluble form of proteins sometimes do not react well with the membrane-bound protein on the living organism, reviewed in [22]. Seeing this unsuccesful inhibiton of *Leishmania* attachment in vivo, we performed *ex vivo* inhibition of *Leishmania infantum* binding to *Lutzomyia longipalpis* midgut by binding experiments. Details of the method are given by Wilson and coworkers [23]. Although we did not find significant reduction of attached parasites in the presence of anti rLuloG, again a tendency to lower parasite numbers was observed (data not shown).

Nevertheless, a key question is what kind of parasite molecule could interact with this glycoconjugate. We may speculate that the parasite receptor for the 45 kDa glycoconjugate is a lectin-like molecule with predicted specificity for GalNAc, mimicking the activity of HPA lectin. Lectin activities and heparin-binding activities were demonstrated in *Leishmania* promastigotes by various authors [24–28]. More recent studies suggest that 65 kDa and 55 kDa heparin-binding proteins from the surface of *L. braziliensis* promastigotes can be involved in parasite adhesion to *Lu. longipalpis* cells through heparan sulphate bridges [29–31]. These heparin-binding proteins were reported to possess metallo-proteinase like activity [31]. As they were not characterized by mass spectrometry, one cannot exclude the possibility that they are in reality identical with some isoforms of the major surface protease, leishmanolysin/gp 63 (reviewed in [32]).

We predict that the parasite receptor would be expressed at a reasonably high level, to ensure that specific binding to the midgut can occur despite the presence of other high copy surface molecules such as LPG. Reviewing current knowledge of the promastigote surface there is a variety of candidate molecules. These include leishmanolysin/gp63, PSA/gp46, mPPG, laminin-binding proteins or the already mentioned heparin-binding proteins, and maybe other unidentified proteins. A potential role for gp63 in parasite attachment has been previously proposed [33, 34]. These studies showed that the monoxenous trypanosomatids *Herpetomonas samuelpessoai* and *Leptomonas* species produce a metallopeptidase that has similar properties to *Leishmania* gp63 and contributes to parasite adhesion to *Aedes aegypti* guts or the *Aedes albopictus* cell line C6/36. These results are consistent with those obtained with *Leishmania* species; using an *ex vivo* by binding assay Jecna and colleagues [11] found that the gp63 of *L. amazonensis* gp63 down-regulated transfectants is functionally important for binding of its promastigotes to the *Lu. longipalpis* midgut.

In conclusion, while the *L. major*-*P. papatasi* work has established an important paradigm, recent evidence indicates that another mechanism of attachment could exist in permissive vectors. The present study has revealed interesting and novel properties of the “19 kDa midgut protein” from *Lu. longipalpis* midguts that can be briefly summarized as follows: (i) it is a mucin-like glycoconjugate with apparent weight of 45 kDa; (ii) it is located on the luminal surface of the midgut; and (iii) recombinant form rLuloG is able to bind to the surface of *Leishmania* cells. Nevertheless, additional studies will be necessary to prove the biological role of the “19 kDa midgut protein”, especially its role as a putative midgut ligand in permissive vectors.

## Conclusions

In this study we characterized a glycoprotein from *Lutzomyia longipalpis* midgut which has been previously proposed as a molecule participating in *Leishmania* binding to the epithelium of the permissive sand flies. We identified that this glycoprotein corresponds to a putative 19 kDa protein with unknown function detected in a midgut cDNA library of *Lu. longipalpis* and named it LuloG. We demonstrate that this mucin-like molecule is GPI-anchored in the membrane of enterocytes and localized it on the luminal side of the midgut. These findings prove accessibility of the mucin for the parasites during blood meal digestion. In line with the putative role of mucin in parasite attachment, we indeed showed binding of a recombinant form of the glycoprotein to the surface of *Leishmania* promastigotes including flagella *in vitro*. However, antibodies raised against recombinat LuloG were not able to block *Leishmania* infection in *Lu. longipalpis in vivo*. In conclusion, our data support strongly the view that LuloG participates in *Leishmania* attachment to the *Lu. longipalpis* midgut and thus is important for parasite transmission.

## References

[1] Dostálová A, Volf P (2012). *Leishmania* development in sand flies: parasite-vector interactions overview. Parasit Vectors.

[2] Killick-Kendrick R, Molyneux DH, Ashford RW (1974). Ultrastructural observations on the attachment of *Leishmania* in the sandfly. Trans R Soc Trop Med Hyg.

[3] Warburg A, Tesh RB, McMahon-Pratt D (1989). Studies on the Attachment of *Leishmania* flagella to sand fly midgut epithelium. J Protozool.

[4] Pimenta P, Turco S, McConville M, Lawyer P, Perkins P, Sacks D (1992). Stage-specific adhesion of *Leishmania* promastigotes to the sandfly midgut. Science.

[5] Pimenta PF, Saraiva EM, Rowton E, Modi GB, Garraway LA, Beverley SM (1994). Evidence that the vectorial competence of phlebotomine sand flies for different species of *Leishmania* is controlled by structural polymorphisms in the surface lipophosphoglycan. Proc Natl Acad Sci U S A.

[6] Kamhawi S, Ramalho-Ortigao M, Pham VM, Kumar S, Lawyer PG, Turco SJ, Barillas-Mury C, Sacks DL, Valenzuela JG (2004). A role for insect galectins in parasite survival. Cell.

[7] Dobson DE, Kamhawi S, Lawyer P, Turco SJ, Beverley SM, Sacks DL (2010). *Leishmania major* Survival in Selective *Phlebotomus papatasi* Sand fly vector requires a specific SCG-encoded lipophosphoglycan galactosylation pattern. PLoS Pathog.

[8] Di-Blasi T, Lobo AR, Nascimento LM, Córdova-Rojas JL, Pestana K, Marín-Villa M (2015). The Flagellar protein FLAG1/SMP1 is a candidate for *Leishmania* –sand fly interaction. Vector-Borne Zoonotic Dis.

[9] Myšková J, Svobodová M, Beverley SM, Volf P (2007). A lipophosphoglycan-independent development of *Leishmania* in permissive sand flies. Microbes Infect.

[10] Svárovská A, Ant TH, Šeblová V, Jecná L, Beverley SM, Volf P (2010). *Leishmania major* glycosylation mutants require phosphoglycans (lpg2-) but not lipophosphoglycan (lpg1-) for survival in permissive sand fly vectors. PLoS Negl Trop Dis.

[11] Jecná L, Dostálová A, Wilson R, Seblová V, Chang KP, Bates PA, Volf P (2013). The role of surface glycoconjugates in *Leishmania* midgut attachment examined by competitive binding assays and experimental development in sand flies. Parasitology.

[12] Volf P, Myšková J (2007). Sand flies and *Leishmania*: specific versus permissive vectors. Trends Parasitol.

[13] Svobodová M, Votypka J, Pecková J, Dvorak V, Nasereddin A, Baneth G (2006). Distinct transmission cycles of *Leishmania tropica* in 2 adjacent foci, northern Israel. Emerg Infect Dis.

[14] Volf P, Volfová V (2011). Establishment and maintenance of sand fly colonies. J Vector Ecol.

[15] Bordier C (1981). Phase separation of integral membrane proteins in Triton X-114. J Biol Chemist.

[16] Myšková J, Votypka J, Volf P (2008). *Leishmania* in Sand Flies: Comparison of quantitative polymerase chain reaction with other techniques to determine the intensity of infection. J Med Entomol.

[17] Warburg A (2008). The structure of the female sand fly (*Phlebotomus papatasi*) alimentary canal. Trans R Soc Trop Med Hyg.

[18] Jochim RC, Teixeira CR, Laughinghouse A, Mu J, Oliveira F, Gomes RB (2008). The midgut transcriptome of *Lutzomyia longipalpis*: comparative analysis of cDNA libraries from sugar-fed, blood-fed, post-digested and *Leishmania* infantum chagasi-infected sand flies. BMC Genomics.

[19] Ramalho-Ortigão M, Jochim RC, Anderson JM, Lawyer PG, Pham VM, Kamhawi S, Valenzuela JG (2007). Exploring the midgut transcriptome of *Phlebotomus papatasi:* comparative analysis of expression profiles of sugar-fed, blood-fed and *Leishmania major*-infected sandflies. BMC Genomics.

[20] Secundino NFC, Nacif-Pimenta R, Hajmova M, Volf P, Pimenta PFP (2005). Midgut muscle network in *Lutzomyia longipalpis* and *Phlebotomus duboscqi* sand flies: spatial organization and structural modification after blood meal. Arthropod Struct Dev.

[21] Reilly RM, Domingo R, Sandhu J (1997). Oral delivery of antibodies. Future pharmacokinetic trends. Clin Pharmacokinet.

[22] Bütikofer P, Malherbe T, Boschung M, Roditi I (2001). GPI-anchored proteins: now you see ´em, now you don’t. FASEB J.

[23] Wilson R, Bates MD, Dostalova A, Jecna L, Dillon RJ, Volf P, Bates PA (2010). Stage-Specific Adhesion of *Leishmania* promastigotes to sand fly midguts assessed using an improved comparative binding assay. PLoS Negl Trop Dis.

[24] Hernandez A, Rodriguez N, Stojanovic D, Candelle D (1986). The localization of a lectin-like component on the *Leishmania* cell surface. Mol Biol Rep.

[25] Mukhopadhyay N, Shome K, Saha A, Hassell J, Glew R (1989). Heparin binds to *Leishmania donovani* promastigotes and inhibits protein phosphorylation. Biochem J.

[26] Schottelius J (1992). Neoglycoproteins as tools for the detection of carbohydrate-specific receptors on the cell surface of *Leishmania*. Parasitol Res.

[27] Svobodová M, Bates PA, Volf P (1997). Detection of lectin activity in *Leishmania* promastigotes and amastigotes. Acta Trop.

[28] Kock N, Gabius H, Schmitz J, Schottelius J (1997). Receptors for carbohydrate ligands including heparin on the cell surface of *Leishmania* and other trypanosomatids. Trop Med Int Heal.

[29] Azevedo-Pereira RL, Pereira MCS, Oliveria-Junior FOR, Brazil RP, Côrtes LMC, Madeira MF (2007). Heparin binding proteins from *Leishmania* (Viannia) braziliensis promastigotes. Vet Parasitol.

[30] de Castro Côrtes L, de Souza Pereira M, da Silva F, Pereira BA, de Oliveira Junior F, de Araújo Soares R (2012). Participation of heparin binding proteins from the surface of *Leishmania* (*Viannia*) *braziliensis* promastigotes in the adhesion of parasites to *Lutzomyia longipalpis* cells (Lulo) in vitro. Parasit Vectors.

[31] de Castro Côrtes LM, de Souza Pereira MC, de Oliveira FOR, Corte-Real S, da Silva FS, Pereira BAS (2012). *Leishmania* (*Viannia*) *braziliensis*: insights on subcellular distribution and biochemical properties of heparin-binding proteins. Parasitology.

[32] Yao C, Donelson JE, Wilson ME (2003). The major surface protease (MSP or GP63) of *Leishmania* sp. Biosynthesis, regulation of expression, and function. Mol Biochem Parasitol.

[33] Pereira FM, Bernardo PS, Dias Junior PFF, Silva BA, Romanos MTV, D’Avila-Levy CM (2009). Differential influence of gp63-like molecules in three distinct *Leptomonas* species on the adhesion to insect cells. Parasitol Res.

[34] Pereira FM, Dias FA, Elias CGR, d’Avila-Levy CM, Silva CS, Santos-Mallet JR, Branquinha MH, Santos ALS (2010). Leishmanolysin-like molecules in *Herpetomonas samuelpessoai* mediate hydrolysis of protein substrates and interaction with insect. Protist.

